# Fat reserve and body condition variation in Argentine black and white tegus: native-invasive comparisons and environmental drivers in Florida

**DOI:** 10.1371/journal.pone.0342916

**Published:** 2026-02-11

**Authors:** Jenna M. Cole, Sergio A. Balaguera-Reina, Melissa A. Miller, Gabriela Cardozo, Margarita Chiaraviglio, Lee A. Fitzgerald, Sergio Naretto, Frank J. Mazzotti

**Affiliations:** 1 Department of Wildlife Ecology and Conservation, Fort Lauderdale Research and Education Center, University of Florida, Fort Lauderdale, Florida, United States of America; 2 Instituto de Diversidad y Ecología Animal (IDEA, CONICET and Universidad Nacional de Córdoba), Córdoba, Argentina; 3 Biodiversity Research and Teaching Collections, Department of Ecology and Conservation Biology, Texas A&M University, College Station, Texas, United States of America; 4 School of Natural Sciences, Macquarie University, Sydney, New South Wales, Australia; University of Missouri, UNITED STATES OF AMERICA

## Abstract

Invasive species impose major ecological and economic costs on ecosystems and countries where introduced. To effectively manage Argentine black and white tegus (*Salvator merianae*) within their invasive range, it is important that management actions are based on species’ biology. We estimated tegu percentage fat and body condition in native (Cordoba, Argentina) and non-native (South Florida, United States) populations and identified biological, temporal, and environmental variables that influence tegu body condition in South Florida. Large adult tegus in Cordoba had larger fat reserves than tegus in South Florida. However, body condition values were highly similar between the native and non-native range throughout the year, showing a well-adapted tegu population to South Florida environmental conditions. Generalized additive mixed models (size estimate = 2.67) showed very strong (p-value < 0.001) to moderate (p-value <0.01) evidence of Julian day, minimum temperature, and percentage fat individually affecting tegu body condition in South Florida (deviance explained 37%). The direction and magnitude of univariate effects varied from positive linear relationship (minimum temperature) impacting body condition up to 18% to negative (Julian day) and positive (percentage fat) monomodal relationships impacting body condition up to 24% and 6%, respectively. Our results provide insights as to how adaptable tegus are physiologically to novel environments and their capability to maintain body condition that is similar to, or better than that of native individuals. These findings can inform management in Florida by identifying seasonal windows when tegus’ activity and condition may make them more susceptible to targeted removal.

## Introduction

The Argentine black and white tegu (hereafter tegu; Teiidae: *Salvator merianae*) is a large-bodied omnivorous lizard native to South America (Brazil, Bolivia, Paraguay, Uruguay, and Argentina) [1,2] known to reach lengths up to 1.2 m and weigh up to 8 kg [3,4]. Tegus were introduced to South Florida more than three decades ago and are currently present throughout the state [5,6] with established populations within Hillsborough, Miami-Dade, Charlotte, and St. Lucie counties [7–9]. South Florida is particularly susceptible to herpetofauna invasions due to a combination of climate, major ports of entry, thriving trade in exotic pets, and occasional destructive weather, which can result in the accidental release of nonnative species from captivity. Tegus pose a significant threat to Florida ecosystems by preying upon eggs of ground nesting species, including American Alligators (*Alligator mississippiensis*) and Gopher tortoises (*Gopherus polyphemus*) [10,11]. Due to their high reproductive potential, broad diet, and ability to survive in diverse habitats, active management is essential to prevent further spread and minimize long-term ecological damage.

Tegus are habitat generalists and occupy a variety of habitats within their native and invasive ranges including rainforest, coastal areas, savannahs, forest clearings, and disturbed areas [1,4,12,13]. Environmental patterns of precipitation, vegetation, and temperature have been found to explain the spatiotemporal distribution of tegus in their native range [1,14]. Due to their generalist behavior [13], vagility, and fecundity [4,15], tegus have proven to be of concern to wildlife management agencies within non-native lands [8]. Within their native range, tegu lizards have been harvested for their skins for at least 70 years, with no evidence of local population extirpation or decline that could be attributed to harvest [15,16], suggesting that the species possesses a remarkable capacity to withstand removals. Therefore, to manage tegus effectively within their invasive range and to curb proliferation of tegus into new areas, management actions should be refined through an increased understanding of the species’ biology. Although many invasive species perform better in introduced areas, often reaching larger sizes, this pattern can vary among species [11]. Here is where understanding how invasive species perform in a new habitat compared to their performance in the native range can direct management actions within the invasive front.

How well have invasive tegus adapted to novel environments? The answer to this question relies in part on knowledge of individual and population-level health indicators, which managers can use to develop actions targeting invasive species. Body condition is a good substitute for measuring whole-organism energy stores, which can be complex and time consuming. Measures of body condition have been used to assess how well individuals are coping with their environment and make inferences about habitat quality [17–22]. Body condition has been linked to several aspects of fitness including reproductive success [23–25], growth [26], survival [27], predator-prey dynamics, habitat quality [28], parasite load [29], probability of detection and capture [30,31], and blood chemistry [32–34]. Moreover, environmental factors such as temperature, rainfall, and land cover could influence energetic reserves related to body condition [24]. Body condition can also influence behavior [35,36]; for example, individuals in poor body condition may engage in risky foraging activity or movement patterns, or be less likely to engage in dispersal, than individuals who are in average or above-average body condition [37–40]. Conversely, dispersal rates may also increase in areas where resources are not available or where competition is high [41].

Understanding how well tegus are performing in Florida compared to native populations is not only of ecological interest—it can directly inform the timing, location, and intensity of control measures as it is a direct indicative of how good/bad invaders tegus are. To better organize efforts aimed at managing and controlling invasive tegus, it is useful to understand how tegu body condition changes under different environmental conditions. In Florida, this knowledge can be used to determine when tegus are most energetically vulnerable (e.g., pre- or post-brumation) and to target trapping and removal efforts when animals are most active above ground or when their movement patterns make them more susceptible to capture. Body condition can be an important factor to the success, dispersal, and further establishment of non-native tegu populations. For example, a Florida study showed that individuals with higher body condition brumate earlier and for longer [42]; managers could adjust removal schedules to focus on lower-condition individuals later in the season, or to prioritize high-condition individuals before they enter refugia.

The primary objective of this study was to compare tegu percentage fat and body condition in native (Cordoba, Argentina) and non-native (South Florida, United States) populations and evaluate biological (percentage fat, size class), temporal (year, Julian day), and environmental (rainfall, temperature, habitat) variables that influence tegu body condition in South Florida.

Tegus are clearly successful invaders in Florida, and their invasive range is expanding. In Argentina, tegus are native to the ecosystems they inhabit, where their life history traits and population dynamics are shaped by long-term ecological relationships, including predation, pathogens, resource availability, and density-dependent factors. Thus, we predicted that invasive tegus in Florida would exhibit an equal or elevated body condition compared to a sample from the native range in Cordoba. We also predicted, based on the species’ biology, that body condition of tegus captured in South Florida is highest in the fall (Sep) before brumation and lowest during spring (Mar-May) because of reproductive activities.

Female tegus, like all squamates, store fat for energy allocation during production of eggs, whereas males expend their energy for reproduction throughout the season while searching for mates, guarding females, and competing with other males [43]. Thus, we predicted females would have higher body condition than males. If confirmed, this pattern could allow targeted female removals prior to nesting, potentially reducing recruitment. We also predicted that adults would have higher body condition than juveniles, suggesting that removal programs may benefit from targeting larger individuals before reproductive output occurs. Finally, we predicted that environmental variables (e.g., temperature, rainfall) have stronger effects on tegu body condition than fixed variables (e.g., habitat type), meaning that managers could anticipate seasonal shifts in vulnerability and adjust control strategies dynamically – though we acknowledge both type of variables may not be mutually exclusive.

## Methods

### Tegu data collection

#### Cordoba, Argentina.

**Fig 1:**
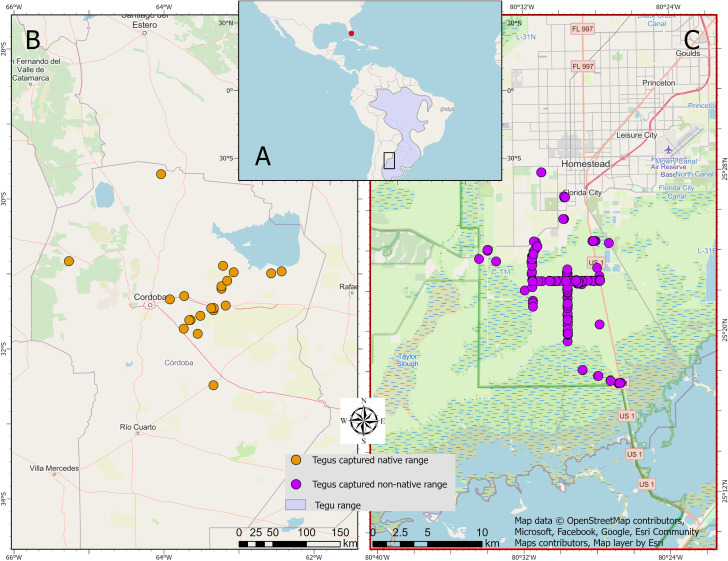
Areas where tegus were captured. **A)** Map of tegu capture locations through their native (Cordoba, Argentina, black rectangle within the purple polygon) and non-native (South Florida, United States, red dot) range. **B)** Captures from Cordoba were part of a project that obtained data from tegus hunted within the framework of the legal skin trade. Roadkill tegus were also included. Note that not all records are included as rural hunters do not have geolocation devices. **C)** Captures coming from South Florida are part of active trapping done to reduce the impact of this invasive species on Florida’s biodiversity. Map was created via ArcGIS pro version 3.4.0. (https://www.esri.com/en-us/arcgis/products/arcgis-pro/overview).

Most tegu specimens from their native range were obtained through the legal skin trade in collaboration with local authorized hunters, and in some instances roadkill, from 2008 through 2012 (Fig 1). Roadkill specimens were fresh, intact, and showed clear signs of having died recently. Our data collection complied Argentine federal law, under the Secretaría de Ambiente y Desarrollo Sustentable as regulated by Resolution No. 2010/2007, Resolution No. 137/2010, and Resolution No. 11/2011. Wild tegu capture was permitted during the study years under provincial hunting regulations, and research permits were issued by the Secretaría de Ambiente y Cambio Climático, Ministerio de Ambiente del Gobierno de Córdoba. Relevant hunting season regulations included Resolutions Nº 327/2008, 750/2009, 1399/2010, 1179/2011, and 865/2012. Since tegus were hunted opportunistically there was no intentional selection of sex by the hunters. At the time of data collection, no Animal Care and Use Committee for wild animals was available at our institution (Universidad Nacional de Córdoba). Nevertheless, we adhered to international standards, following guidelines such as those of the AVMA (2007). Euthanasia was conducted through cranial concussion followed by decapitation and pithing, in accordance with the AVMA Guidelines on Euthanasia available at the time of the study [44].

#### South Florida, United States.

Tegus from their invasive range were captured through removal efforts (live trapping) performed by the University of Florida, Florida Fish and Wildlife Conservation Commission (FWC), United States Geological Survey (USGS), and National Park Service (NPS) from 2014 through 2019. Tegus were humanely euthanized within 24 hours of capture to minimize distress. Euthanasia was conducted using captive bolt or firearm immediately followed by pithing, following AVMA standards [44] and frozen until necropsy. Research was conducted under animal research protocols approved by the University of Florida Institutional Animal Care and Use Committee (ARC #001-10FTL, ARC #001-11FTL, ARC #012-11FTL, IACUC #201408432, IACUC #201708432) in compliance with the ARRIVE guidelines [45] and performed in accordance with relevant guidelines and regulations. All tegus captured came from Miami-Dade County mostly from the Southern Glades Wildlife and Environmental Area and in locations north, east, and west of it, including the Homestead Air Reserve Base, plant nurseries, agricultural lands, residential dwellings, and the surrounding areas of the C-111, C-111E, and Aerojet canals, South Dixie Highway, and Card Sound Road.

#### Data collection.

Tegus were examined for general health by visually inspecting all internal organs and the body exterior for abnormalities or deformities that may affect body mass, body length, or fat mass. Data were collected both from live specimens and again after sacrifice. We measured total length (TL), snout-vent length (SVL), and total body mass using a flexible measuring tape and a Pesola precision spring scale appropriately sized for the tegu being measured. We acknowledge the potential for minimal mass loss when weighing organs and performed these measurements as quickly as possible during necropsy. Coelomic wet-fat mass was obtained by removing and weighing abdominal fat bodies to the nearest 0.0001 gr. Percentage fat mass was calculated by dividing wet-fat mass by total body mass times 100. Given the retrospective nature of this study, it is important to acknowledge the differences in methodology and collection timing between sampling native and nonnative tegus. However, the variables measured and analyses conducted to compare body condition are unlikely to be biased by the sampling method as we compared tegus captured year-round, considering the reproductive cycle/activity patterns derived from the seasonality of the species.

To account for differences in size, tegus were assigned to one of three size-class groups based on snout-to-vent length (SVL) measurements: hatchlings and early juveniles with SVLs up to 20.2 cm, late juveniles to reproductive-sized adults with SVLs measuring 20.2–30.0 cm, and large adults SVL measuring 30.0 cm and greater. These groups were defined by looking for breakpoints across the relationship between mass and SVL based on linear regression models as described by McCaffrey et al. [46]. We only used large adults for inter-population comparisons (see results) as this was the only consistent group with large sample size in both native and non-native populations across time.

### Body condition assessment

Body condition is usually expressed as an index based on measurements of length and mass, such as Fulton’s K [19]. Fulton’s K is a ratio calculated by taking body mass (W) divided by body length (L) cubed **(**K = W/L^3^) and was first employed to assess body condition in fisheries science [19,47]. Fulton’s K was identified as the best performing metric for tegus outperforming 10 other methods commonly used [46]. Body condition for tegus in the native and non-native range was calculated and compared using Fulton’s K index. Body condition outliers were removed using interquartile ranges -IQR [48] calculated in R version 4.3.1 [49] as well as tegus with physical abnormalities (missing limbs, regenerated tails, missing tails, and crooked spines) as these can influence Fulton’s K slope [42].

We tested the effect of variables such as sex, size group, and seasonal periods in both native [brumation emergence (August), reproductive (September – December), post-reproductive (January-February), entrance into brumation (March), and brumation (April-July) [43,50]] and non-native [brumation emergence (February), reproductive (March-May), post-reproductive (June-August), entrance into brumation (September), and brumation (October-January) [51,52]] range on Fulton’s K values via Wilcoxon rank sum and Kruskal-Wallis tests. Dunn pairwise test using Bonferroni correction was used to define pairwise differences between size groups.

Critical values (p-values) for statistical evidence were defined as graded evidence (very strong -p-value < 0.001, strong -p-value < 0.01, moderate -p-value < 0.05, weak -p-value < 0.10, and little to no evidence -p-value > 0.10) and interpreted in conjunction with relevant information about data and statistics as recommended by Muff et al. [53–55], Lakens [56], Hartig and Barraquand [57], and Amrhein and Greenland [58], providing better clarity about the meaning of our statistical results in the context of our study.

### Factors influencing body condition in the non-native range

We assessed the effect of physiological (percentage fat), temporal (year, Julian day), environmental (mean rainfall and temperature and monthly minimum maximum temperature), and habitat type (prairies and bog, marshes, freshwater forested wetlands, or urban) variables on tegu body condition in South Florida via generalized additive mixed models (GAMM) for large datasets (*bam* function, “mgcv” package) [59]. GAMMs are a flexible modeling framework that accommodates non-linear relationships and includes random effects. Temporal variables were obtained from capture date. Mean monthly weather data were gathered from the Florida Automated Weather Network (https://fawn.ifas.ufl.edu/) Homestead station. Habitat type was determined for each capture location using the Florida Cooperative Land Cover Map (CLC), Version 3.5 [60]. We modified CLC to better describe the habitat where tegus were captured by removing polygons labeled as canal, transport, and vegetated berm and replacing them with the adjacent habitat, which more appropriately described the area where captures occurred. CLC polygons were rasterized to 1 m x 1 m pixels via polygon to raster tool in ArcGIS pro 3.2.1 [61], clipped, and values were extracted to the capture locations of each tegu. Once assigned, habitat was then categorized based on the CLC classification system [60].

Selection of covariates and determination of their importance by groups was done using a wrapper method (random forest classification algorithm) via the Boruta package [62]. The algorithm was trained 1,000 times and variable importance was defined based on the mean decrease accuracy. Only variables with a Z-score higher than the shadow (shuffled) variables were selected as relevant. Once relevant variables for tegu body condition were defined, we parametrized a set of 6 GAMM models and ran them under the Gaussian family distribution error with identity link (Table 1) using Fulton’s K as a response variable, and array of predictors in different combinations of parametric, univariate, bivariate, factor smooths, and random effects. We selected the best (most parsimonious) model structure based on the Akaike Information Criteria (AIC; the lowest value; *aic* function) and assessed whether the information provided by the best model differed from the others via analysis of deviance (*anova* function) for *gam*.

**Table 1:** Generalized additive mixed model (GAMM) parametrization used to assess the effect of environmental and physiological variables on tegu body condition in South Florida, United States, estimated via Fulton’s K.

Model	AIC	ΔAIC	DE (%)
Fulton’s K ~ s(Julian) + s(Rainfall) + s(Mean Temp) + s(Min Temp) + s(Max Temp) + s(Percentage fat) + ti(Julian, Percentage fat) + ti(Rainfall, Percentage fat) + ti(Mean Temp, Percentage fat) + ti(Min Temp, Percentage fat) + ti(Max Temp, Percentage fat) + s(Year, bs = ‘re’) + s(Size Class, bs = ‘re’) + s(Habitat Category, bs = ‘re’)	128.05		35.3
*Fulton’s K ~ Habitat Category + s(Julian) + s(Rainfall) + s(Mean Temp) + s(Min Temp) + s(Max Temp) + s(Percentage fat) + s(Julian, Year, bs = ‘fs’) + s(Rainfall, year, bs = ‘fs’) + s(Mean Temp, Year, bs = ‘fs’) + s(Min Temp, Year, bs = ‘fs’) + s(Max Temp, Year, bs = ‘fs’) + s(Percentage fat, Year, bs = ‘fs’) + ti(Julian, Percentage fat) + ti(Rainfall, Percentage fat) + ti(Mean Temp, Percentage fat) + ti(Min Temp, Percentage fat) + ti(Max Temp, Percentage fat) + s(Size Class, bs = ‘re’)*	*128.88*	*0.83*	*37.3*
Fulton’s K ~ s(Julian) + s(Rainfall) + s(Mean Temp) + s(Min Temp) + s(Max Temp) + s(Percentage fat) + s(Julian, Habitat Category, bs = ‘fs’) + s(Rainfall, Habitat Category, bs = ‘fs’) + s(Mean Temp, Habitat Category, bs = ‘fs’) + s(Min Temp, Habitat Category, bs = ‘fs’) + s(Max Temp, Habitat Category, bs = ‘fs’) + s(Percentage fat, Habitat Category, bs = ‘fs’) + ti(Julian, Percentage fat) + ti(Rainfall, Percentage fat) + ti(Mean Temp, Percentage fat) + ti(Min Temp, Percentage fat) + ti(Max Temp, Percentage fat) + s(Year, bs = ‘re’) + s(Size Class, bs = ‘re’)	130.68	2.63	36.3
Fulton’s K ~ s(Julian) + s(Rainfall) + s(mean Temp) + s(Min Temp) + s(Max Temp) + s(Percentage fat) + s(Julian, Year, bs = ‘fs’) + s(Rainfall, Year, bs = ‘fs’) + s(Mean Temp, Year, bs = ‘fs’) + s(Min Temp, Year, bs = ‘fs’) + s(Max Temp, Year, bs = ‘fs’) + s(Percentage fat, Year, bs = ‘fs’) + ti(Julian, Percentage fat) + ti(Rainfall, Percentage fat) + ti(Mean Temp, Percentage fat) + ti(Min Temp, Percentage fat) + ti(Max Temp, Percentage fat) + s(Habitat Category, bs = ‘re’) + s(Size Class, bs = ‘re’),	131.97	3.92	36.4
Fulton’s K ~ Habitat Category + s(Julian) + s(Rainfall) + s(Mean Temp) + s(Min Temp) + s(Max Temp) + s(Percentage fat) + s(Julian, Year, bs = ‘fs’) + s(Rainfall, Year, bs = ‘fs’) + s(Mean Temp, Year, bs = ‘fs’) + s(Min Temp, Year, bs = ‘fs’) + s(Max Temp, Year, bs = ‘fs’) + s(Percentage fat, Year, bs = ‘fs’) + ti(Julian, Percentage fat) + ti(Rainfall, Percentage fat) + ti(mean Temp, Percentage fat) + ti(Min Temp, Percentage fat) + ti(Max Temp, Percentage fat) + s(Size Class, bs = ‘re’)	141.16	13.11	35.7
Fulton’s ~ s(Julian) + s(Rainfall) + s(Mean Temp) + s(Min Temp) + s(Max Temp) + s(Year, bs = ‘re’) + s(Size Class, bs = ‘re’) + s(Habitat Category, bs = ‘re’	615.35	487.3	17.6

Models are ordered from the lowest Akaike Information Criteria (AIC) to the highest, presenting also the deviance explained (DE) by the predictors. Although the first model had the lowest AIC there was no evidence of differences between the first and second model. Therefore, we chose model 2 (italicized) as the best model due to it having the highest deviance explained.

The best model (see results) was parametrized including habitat type as a parametric term, all covariates as univariate thin plates splines (‘tp’), the most relevant variable selected plus all other covariates as bivariate tensor interactions (‘ti’), all variables interacting with year as a factor smooth (‘fs’), and size class as a random effect (‘re’) to account for any variability that might be present in the data due to factors that are not explicitly included in the model. We ran the best model under Gamma family distribution error with log link (fifth model on Table 1) to assess whether the distribution error would improve model quality. We also ran a sixth model excluding percentage fat to evaluate the weight of this variable on modeling. Finally, we tested whether the selected theoretical distribution fit our models via QQ and residual plots (*gam.check* function) [63].

We used the default basis complexity (k) for all models and checked whether this value was enough for each parameter via the *gam.check* function. Models were run using the fast restricted maximum likelihood (fREML) and the double penalty approach was selected (select = TRUE) allowing us to penalize the null space without dropping any covariate within post processing [64]. Finally, the best model was plotted using the *draw* function from the “Gratia” package [65] and the *plot_prediction* function from the “marginaleffects” package [66].

## Results

We processed a total of 1,634 tegus from South Florida (n = 748 female, 886 male) and 628 tegus from Cordoba (265 females, 363 males; Table 2). Most tegus captured in South Florida were hatchlings-early juveniles and late juvenile-early adults (90.9%) whereas most of the tegus captured in Cordoba were large adults (97%). Intrapopulation analysis showed no evidence of differences in SVL (W = 322642, p-value = 0.36) and mass (W = 321136, p-value = 0.28) between sexes in tegus captured in South Florida, but it did show very strong evidence of differences in percentage fat by sex (W = 64230, p-value = 0.0002, females 1.3 ± 1.3%, males 1.1 ± 1.5%). In contrast, we found very strong evidence (all p-values < 0.001) of differences in size and mass by sex in tegus captured in Cordoba having larger and heavier males (SVL: 38.7 ± 4.4 cm; weight: 1,928.5 ± 694.3 g) than females (SVL: 37.4 ± 3.1 cm; weight: 1,702.8 ± 500.4 g), but females having larger fat reserves (4.1 ± 2.6%) than males (2.1 ± 1.7%). Overall, smaller tegus had lower Fulton’s K body condition index compared to larger tegus in both native and non-native range ranging from 2.9 to 3.2 in South Florida and 2.7 to 3.2 in Cordoba (Table 2).

**Table 2:** Morphometric data collected from Argentine black and white tegus captured in their native (Cordoba, Argentina) and invasive (South Florida, United States) range.

Gr	Ar	N	F	M	SVL (cm)	W (g)	MFK	MCFW (g)	MPF (%)	N nec
HE	SF	403	208	195	17.0 ± 2.4	153 ± 62.2	2.9 ± 0.3	1.6 ± 2.7	0.9 ± 1.3	151
	Cor	2	0	2	19.5 ± 0.7	200 ± 14.1	2.7 ± 0.1			0
LJA	SF	1081	468	613	24.8 ± 2.6	488 ± 167	3.1 ± 0.3	5.9 ± 8.8	1.1 ± 1.4	453
	Cor	17	5	12	28 ± 1.6	647 ± 129	2.9 ± 0.4	7.2 ± 5.9	1.0 ± 0.8	12
LA	SF	150	72	78	33.4 ± 2.9	1,228 ± 360	3.2 ± 0.3	22 ± 23.8	1.8 ± 1.6	63
	Cor	609	260	349	38.5 ± 3.5	1,872 ± 599	3.2 ± 0.4	56.0 ± 50.2	2.9 ± 2.3	547
All	SF	1634	748	886	23.7 ± 5.2	473.4 ± 330.2	3.1 ± 0.3	6.5 ± 11.7	1.1 ± 1.4	667
	Cor	628	265	363	38.2 ± 4	1,833.3 ± 639.4	3.2 ± 0.4	54.9 ± 50.1	2.8 ± 2.3	559

Native tegus collected (Cordoba -Cor; 2008–2012) and invasive tegus collected (South Florida -SF; 2014–2019) range grouped by size (HE = hatchling-early juveniles, LJA = late juveniles to reproductive-sized adults, LA = large adult) as defined by McCaffrey et al. [46]. Gr = Size group, Ar = area, N = total tegus measured, F = female, M = male, SVL = mean snout-vent length, W = mean weight, MFK = mean Fulton’s K, MCFW = mean celomic fat weight, MPF = mean percentage fat, N nec = total tegus necropsied, and fat measurements taken. Values are expressed as mean ± standard deviation.

Interpopulation analysis including large adults-only showed very strong evidence (W = 12394, p-value < 0.001) that, on average, tegus captured in Cordoba had larger fat reserves (mean percentage fat = 2.8 ± 2.3%) than tegus captured in South Florida (1.8 ± 1.6%). This result was mainly driven by sex as we found very strong evidence of females (n = 260, 4.1 ± 2.6%) and males (n = 349, 2.1 ± 1.7%) from Cordoba almost doubling fat reserves of females (n = 72, 2.4 ± 1.7%) and males (78, 1.2 ± 1.4%) from South Florida (W = 1940, p-value < 0.001). Interestingly, interpopulation analysis using body condition of large adults-only showed no evidence of differences between tegus captured in Cordoba and South Florida (W = 15671, p-value = 0.25) nor differences by sex (female = 3.17 ± 0.45 and 3.20 ± 0.31, male = 3.17 ± 0.40 and 3.22 ± 0.26).

**Fig 2:**
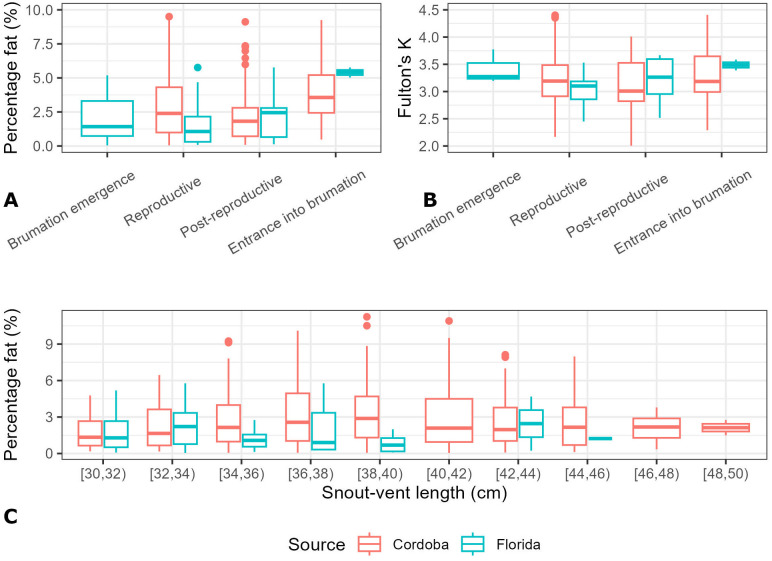
Argentine black and white tegu percentage fat. **(A) and Fulton’s K (B) calculated for tegus captured in the native (Cordoba, Argentina) and non-native (South Florida, United States) range by seasonal period.** Plot C shows that although sampling from Cordoba had more large adult tegus than South Florida, the representation of sizes captured was similar across both populations.

Seasonal comparison showed very strong evidence of differences in percentage fat between native and non-native tegus during reproductive season [Cordoba (n = 467; 2.91 ± 2.30%), Florida (n = 41; 1.44 ± 1.40%), p-value = < 0.001; Fig 2]. However, these differences disappear when tegus enter the post-reproductive season [native (n = 67; 2.26 ± 2.05%), invasive (n = 17; 2.16 ± 1.64%), p-value = 0.85] as well as when tegus enter into brumation [Cordoba (n = 12, 3.91 ± 2.49%), Florida (n = 2, 5.39 ± 0.52%), p-value = 0.44]. We could not test the brumation emergence period (native range = August, non-native range = February) due to lack of tegus captured in Cordoba at this time. As in the case of percentage fat, body condition analysis showed moderate evidence of differences between native and non-native tegus during the reproductive season [Cordoba (n = 467; 3.19 ± 0.40), Florida (n = 41; 3.04 ± 0.27), p-value = 0.01; Fig 2]. However, these differences disappear when tegus enter the post-reproductive season [native (n = 67; 3.11 ± 0.45), invasive (n = 17; 3.22 ± 0.37, p-value = 0.40] as well as when tegus enter into brumation [Cordoba (n = 12, 3.36 ± 0.59), Florida (n = 2, 3.49 ± 0.14), p-value = 0.79].

### Factors influencing body condition in the non-native range

Variable selection analysis showed that most of the variables were important (performed better than random) for body condition (Fulton’s K) except for habitat type and sex (Z-score = −0.29 and −0.05, respectively). The most important variable was percentage fat (Z-score = 42.6) followed by Julian day (Z-score = 11.5), minimum and maximum temperature (Z-score = 7.9 and 6.9), year (Z-score = 6.7), mean temperature and rainfall (Z-score = 6.5, each).

**Fig 3:**
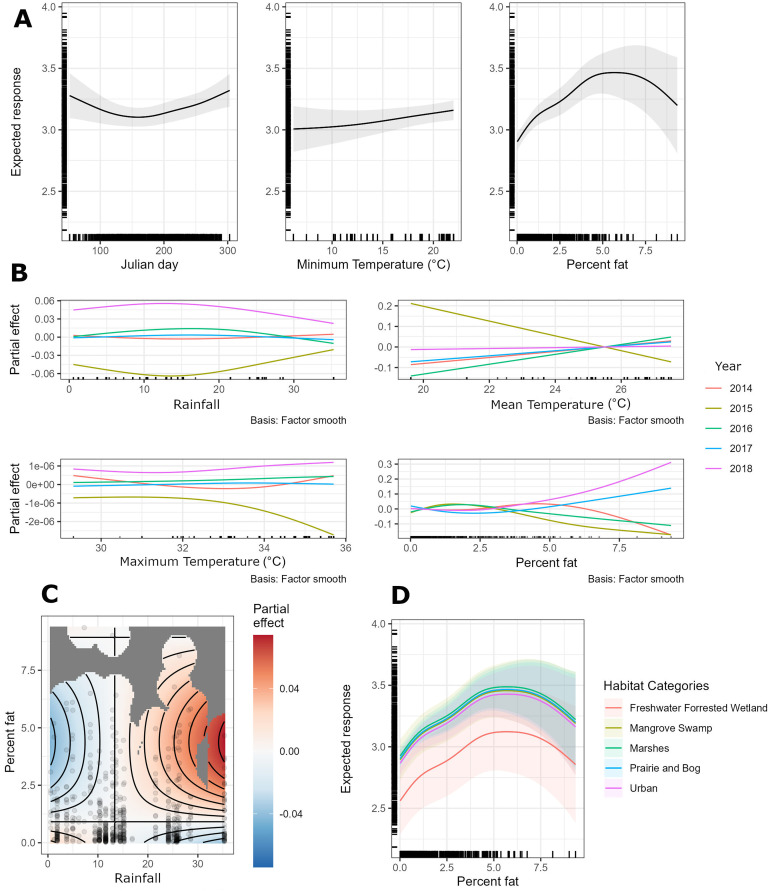
Univariate and bivariate partial effects (all predictors fixed at the mean value except for the variable of interest) and expected responses of tegu body condition in South Florida, United States, based on predictors that showed at least weak evidence of an effect. **3a.** Expected responses of tegu body condition based on change of Julian day, minimum temperature, and percentage fat. **3b.** Partial effects of rainfall, mean temperature, maximum temperature, and percentage fat by year. **3c.** Partial effect of the interaction of two variables rainfall and percentage fat. **3d.** Expected response of tegu body condition based on change of percentage fat by habitat type. Notice how variables such Julian day and percentage fat have the largest impact (change in magnitude) in body condition, and predictors such as Maximum temperature the lowest.

The best GAMM (Gaussian distribution identity link, deviance explained by the model 37.3%, size estimate = 2.67) showed very strong to moderate evidence of Julian day, minimum temperature, and percentage fat individually affecting tegu body condition in South Florida (Fig 3a). The direction and magnitude of univariate effects varied from linear positive relationship (minimum temperature) impacting body condition up to 0.2 units when temperature change around 10°C to inverse monomodal (Julian day) and positive monomodal (percentage fat) impacting body condition up to 0.2 and 0.5 units, respectively when in reproductive season (around May) and when percentage fat changes from 0 to 5% (Fig 3a).

Among years, other variables such as rainfall (mainly in 2015 and 2018), mean temperature (mainly in 2015 and 2016), and maximum temperature (mainly in 2015) influenced tegu body condition (Fig 3b). From those, mean temperature had the largest impact on tegu body condition dropping values up to 0.3 units when increasing from 20 to 26° C in 2015. It is important to note that although maximum temperature came out as a relevant variable when factored by year, the magnitude (change in body condition) was negligible (less than 0.001 body condition units). In 2015 there was a negative effect of rainfall on tegu body condition with the opposite occurring in 2018. Differences between these years were also detected when analyzing percentage fat, explaining a decreasing body condition in 2015 up to 0.1 units and increasing body condition in 2018 up to 0.3 units.

Bivariate interactions showed only rainfall weakly affecting body condition when combined with percentage fat with high rainfall values (around 30 cm monthly) and percentage fat values around 5% providing an increase in body condition values of up to 0.04 units (Fig 3c). Finally, all habitat types showed strong positive linear effect on tegu body condition having an impact of body condition up to 0.3 units except for fresh forested wetland where we got an impact of 0. When relating the most important variable (percentage fat) with habitat categories, we observed that tegus captured in most habitats had body condition values ≥ 3 units except those captured in freshwater forested wetlands, where most tegus sustained body condition values < 3 units (Fig 3d).

## Discussion

Invasive species management is a priority globally due to the deleterious effects introduced species have on native wildlife and ecosystems [67,68]. To increase efficacy and optimize resources, management strategies aimed at mitigating effects of invasive species are best informed by an understanding of the species’ biology in their native and non-native range. Our study demonstrates a novel comparative analysis of Argentine black and white tegus from their native (Cordoba, Argentina) and non-native (South Florida, United States) range, showing that although tegus in their native range can accumulate higher coelomic fat, there is no evidence of overall differences in body condition, showing tegus in Florida are doing as well as they do in their native range (Fig 2). This suggests that invasive tegus in Florida are physiologically capable of maintaining body reserves comparable to native populations, which helps explain their persistence and spread. For managers, this means that Florida tegus remain robust and active for much of the year—key information for determining when trapping will be most effective.

The low variation of body condition (Fulton’s K) between large adult tegus from South Florida and Cordoba, with values above 3 in both regions, indicates consistently good condition [46]. In Cordoba, many individuals were likely harvested before nesting season while still carrying large fat reserves, which could explain higher native-range values. In Florida, similar pre-brumation conditions suggest that trapping just before this period could intercept individuals before they retreat underground, aligning removal efforts with peak capture probability.

We described an interesting temporal pattern in fat reserves and body condition of tegus, with similar values of percentage fat and Fulton’s K in both native and non-native tegus prior to brumation. The notably lower percentage fat during the reproductive stage in Florida implies that tegus may be more food-motivated during this time—providing a potential opportunity for baited traps to be more effective. Targeting trapping during the reproductive period may therefore maximize encounter and capture rates. Our data showed that most of the large adult tegus captured in Florida happened throughout the reproductive season (Mar-Apr, 41 out of 63), showing that tegus are more likely prompt to incur risky behaviors due to low body condition/fat reserves.

There were more exacerbated differences observed during the reproductive periods. These differences were more marked in percentage fat than in Fulton’s K values, but the pattern was similar. These trends in fat reserves and body condition values across the annual life cycle suggest that tegus in South Florida enter brumation in similar conditions to tegus in their native range. This implies tegus in South Florida are well adapted to their new environment. However, the low percentage fat accumulated in the reproductive period in South Florida shows tegus in their invasive range could be either allocating more energy for reproduction than tegus in the native range or having more difficulty building up fat reserves across this period. Another explanation for the lack of fat accumulation could be related to prey availability or prey quality in the invasive range. It agrees with fat body mass reduction observed in association to follicle development and the resulting female emaciation [69]. A third hypothesis could be that tegu metabolic rate in the non-native range could be higher than in the native range due to the increased and prolonged environmental temperature (from April to August), which could limit the amount of fat reserves the species can produce and retain in the reproductive period. These findings are relevant to managers in Florida as it shows the reproductive and post-reproductive periods could be the best time to deploy baited traps as tegus may be more actively seeking food resources.

In native populations, females had higher fat reserves than males, which reflects reproductive requirements and the importance of fat reserves for egg production [69,70]. In the Florida population, despite statistical significance, fat reserves were virtually identical with only 0.2% difference between male and females, so the biological relevance could be seen as minimal, if not negligible. Since fat reserves were nearly identical between sexes, suggesting that sex-specific trapping strategies may not yield significant benefits; instead, managers may achieve more by focusing on seasonal timing and environmental cues that influence activity. Lower levels of fat reserves observed in male tegus may indicate a contribution of other tissues (e.g., muscle) to male body [71–73].

Currylow et al. [42] demonstrated that tegus with higher body condition went into brumation earlier, for longer periods of time, and maintained higher fat stores than tegus with lower body condition in South Florida. Similarly, Fitzgerald et al. [44] reported that larger individuals disappeared from trap arrays during cooler months, meanwhile smaller individuals remained active during those months across the native range. This appears similar to our findings, with large adult tegus disappearing from traps after September and maintaining higher fat reserves than smaller tegus. Lizard body size has previously been linked to activity patterns and habitat selection [4,74]. In their native range tegu activity is influenced by seasonal changes in temperature [1] a pattern that is not as apparent in South Florida. Further research aimed at identifying physiological mechanisms driving behavior and activity could be useful for developing methods for targeting reproductively active individuals in the invasive range. For trapping programs, this means that trap deployment should not only account for seasonality but also for size-specific activity patterns, with efforts early in the season targeting large adults and later periods focusing on juveniles and subadults.

A primary reason for differences in datasets between Cordoba and South Florida is the original purpose of the sampling. In Cordoba, sampling was focused on sexually mature individuals for research on reproductive biology [15,75–77]. In contrast, sampling in Florida was aimed at removing all individuals regardless of body size [46]. Despite these different objectives, our results offer valuable insights from a broader perspective. Beyond body condition, other key biological parameters, such as minimum reproductive size, number of offspring, and reproductive frequency, could be compared to enhance our understanding of invasion processes. Both sampling efforts are linked to conservation: in the native zone, the goal is to conserve a commercially exploited species [78], while in the invaded zone, the focus is on population control. This underscores the importance of collaboration between researchers, as studies like this can inform conservation tools in both contexts. On one hand, these tools can improve management strategies in the invaded zone; on the other, they can shed light on how the species adapts to new environments and helps predict responses to future environmental changes in its native habitat.

### Factors influencing body condition in the non-native range

Most of the variables assessed in the present study that showed an effect on tegu body condition in South Florida were either parametric (linear), univariate (non-linear), univariate factored by habitat category or year, or bivariate (Fig 3) Together, these variables accounted for up to 37% variation in tegu body condition. It is plausible that other variables not measured in this study, such as metabolic rate, resource availability, size of established home range, movements, and reproductive effort also influence body condition of tegus. Percentage fat was a relevant indicator of body condition explaining by itself almost half of the variation in body condition across tegus in South Florida. However, one important point of this analysis is that all other variables still explain half of the variation found in the response, meaning that body condition should not be completely assumed to be described by fat reserves but as a cumulative effect of physiological and environmental variables.

Our approach allowed us to identify percentage fat and Julian day, and to a lesser extent minimum temperature as the most important variables affecting tegu body condition in South Florida. These variables can drive changes in body condition above and below mean values as 1) tegu’s percentage fat moves between 0 and 5%, 2) tegus moves from pre or post brumation to reproductive and post-reproductive season, or 3) monthly minimum temperature change from below 10°C to above 20°C. Variables such as rainfall and maximum temperature can also affect body condition when interacting with other variables such as percentage fat or factored by year. For example, rainfall may influence body condition by altering prey availability or foraging behavior, especially during critical periods like post-brumation emergence or reproductive investment. Interestingly, our top model showed that body condition improves more in wet years (e.g., 2018 up to 0.3 units) than it declines in dry years (e.g., 2015 only impacted tegu body condition up to 0.1 units). This highlights the possibility that favorable moisture conditions enhance resource availability and body condition. It is possible that the effects of environmental variables on tegus are context-dependent, and that interactions between temperature, rainfall, and energy stores may collectively shape seasonal and interannual variation in body condition.

Freshwater forested wetlands stood out as the habitat type where we found the lowest body condition of tegus in South Florida. We speculate that tegus captured in freshwater forested wetlands may be experiencing lower body condition due to reduced access to resources or greater physiological demands. These areas may represent marginal or suboptimal foraging environments. Alternatively, tegus in poor body condition may be more likely to be found in these areas due to behavioral shifts, competitive displacement, or changes that otherwise lead lower-condition individuals to occupy this habitat. The specific mechanism remains unclear, but the association between this habitat type and reduced body condition suggests a mismatch between tegu energetic needs and what this environment offers.

We did not expect to find that there is a limit to how much percentage fat can contribute to body condition. That is, when the percentage fat reaches around 7%, body condition declines. Fat stores, particularly celomic fat stores, are depleted as eggs are formed in female tegus [69,70]. Body fat is also stored in muscle, liver and tail tissues [71,72]. We did not investigate the contribution of muscle, water, or other sources of matter to overall tegu body condition. The contribution of other tissue to body condition can be seen when comparing native and Florida tegu body condition relative to measure of actual fat and percentage fat. While there is no indication that tegus in South Florida were not in good condition, our sample of tegus from Cordoba were larger, heavier, and fatter (again, large individuals were targeted by commercial hunters in Cordoba). It is possible that other tissues may contribute relatively more to tegu body condition in Florida than in the native range, and body condition of male and female tegus in the Florida population may also be influenced by differences in morphology between the sexes.

Tegus are a successful invasive species, particularly in South Florida, and populations exist throughout the state and in other southern states [9,79,80]. The body condition of tegus in South Florida may provide an indication of what “ideal” invasive tegu body condition looks like in optimal conditions. We would expect tegu body condition to decrease in cooler and drier environments, corresponding to a shorter activity season. Body condition is directly related to reproductive success and dispersal, which are particularly relevant when assessing invasions. Our results and future research on body condition of tegus may provide further insight and understanding of how tegus are responding to habitat, seasonal, and environmental differences throughout their invasive and native range.
